# Modifying Effect and Mechanism of Polymer Powder on the Properties of Asphalt Binder for Engineering Application

**DOI:** 10.3390/polym15244659

**Published:** 2023-12-10

**Authors:** Wensheng Zhao, Xiaolong Sun, Zhixin Ou, Zhijian Li, Zhisheng Liu, Xiao Qin

**Affiliations:** 1 National Engineering Research Center of Highway Maintenance Technology, Changsha University of Science & Technology, Changsha 410114, China; zws197009@outlook.com (W.Z.); xls1998@gdut.edu.cn (X.S.); 2 School of Traffic & Transportation Engineering, Changsha University of Science & Technology, Changsha 410114, China; 3 School of Civil and Transportation Engineering, Guangdong University of Technology, Guangzhou 510006, China; ozx2112109146@outlook.com (Z.O.); lzj200003@outlook.com (Z.L.); 4 Key Laboratory of Road and Traffic Engineering, Ministry of Education, Tongji University, Shanghai 201804, China; 1911552@tongji.edu.cn; 5 School of Transportation and Civil Engineering and Architecture, Foshan University, Foshan 528000, China

**Keywords:** road engineering, polyurea, modified asphalt, property, modification mechanism

## Abstract

For achieving the better modifying effect of polyurethane on asphalt pavement materials, the PUA powder modifier was prepared with fine grinding at the glass transition temperature, and polyurethane-modified asphalt (PUA-MA) with different dosages of modifier was prepared. The impact of the PUA on the physical properties of asphalt binder was studied. The modifying mechanism of PUA on asphalt was explored by investigating the thermal performance and chemical composition of asphalt (thermogravimetric analysis, differential scanning calorimetry test, and Fourier transform infrared spectroscopy). The micrograph of the interactive interface was characterized by scanning an electron microscope. Furthermore, the rheological properties of PUA-MA were also investigated and analyzed. The results indicated that the PUA had a dense structure with few pores on the surface. After mixing with asphalt, it altered the asphalt’s internal structure via physical fusion and chemical reaction (carbamate formation). PUA improved the thermal stability of asphalt, enhanced the asphalt’s thermal decomposition temperature, and further reduced the thermal mass loss while decreasing the glass transition temperature. The addition and dosage increase in the PUA modifier significantly improved the softening point, viscosity, complex shear modulus, and rutting factor of asphalt. Also, the PUA could improve the elastic recovery ability of asphalt and enhance the rutting resistance of asphalt at high temperatures. However, the crack resistance at low temperatures was not effectively improved (ductility and penetration decreased). When the dosage was 6–9%, PUA-MA had the best high-temperature performance, but asphalt showed poor low-temperature performance at this dosage. This study provides a theoretical reference for popularizing and applying polyurethane as an asphalt modifier in road engineering.

## 1. Introduction

Due to the long-term coupling effect of vehicle load and environment, the service performance and durability of asphalt pavement deteriorated gradually, leading to the development of road failures such as rutting, cracking, and hugging, which promoted a negative impact on road traffic safety and the service life of asphalt pavement [1,2]. Conventional asphalt pavement confronted a significant challenge in satisfying the growing demands of modern traffic [3]. Modifying asphalt pavement materials using polymer materials was one of the primary techniques for enhancing pavement performance. Currently, the commonly used polymer modifiers mainly include styrene-butadiene-styrene (SBS), ethylene-vinyl acetate (EVA), low-density polyethylene (LDPE), and elastomer modifiers, et al. [4,5,6]. EVA, LDPE, and SBS were the typical additives for asphalt physical modification. Due to the substantial difference in density, relative molecular mass, and solubility between the modifiers and asphalt, the crosslinking network structure was mainly formed by molecular adsorption with asphalt. Mixing created the physical blending and melting effect without significant chemical reactions. Consequently, polymer segregation might be more likely to occur during the manufacturing, storage, and construction of modified asphalt pavement materials [7,8,9]. Elastomer modifiers were one type of chemical modifier. Unlike the physical asphalt modifiers, elastomer materials would enhance the technical properties of pavement materials by reacting with the active functional groups in asphalt and modifying the asphalt’s molecular structure. Moreover, the obtained modified asphalt had better storage stability, and the enhancement effect of asphalt performance could be effectively ensured [10,11].

Elastomer material blocks copolymers of soft and hard segments composed during the polymerization process. Due to its excellent chemical stability, aging resistance, wear resistance, and high elasticity, it was widely used in coatings, electronic components, civil engineering, automobile manufacturing, and medical instrumentation [12,13]. Existing elastomer materials generally comprise polyolefin (TPO), polyurea (PUA), polyurethane (TPU), polyamide (TPAE), polyester (TPEE), polyvinyl chloride (TPVC), and polysiloxane (TPSE). Elastomer material has been explored as an asphalt pavement material modifier because of its excellent functional potential [14]. Polyurethane polyamide and polyurea elastomers consist of crystalline hard segments with a high melting point (polyamide, isocyanate) and noncrystalline soft segments (polyester or polyether) [15]. The high performance depends on the characteristics of the hard segment and the length of the soft and hard parts. Due to its hard segment, the elastomer material had exceptional tensile strength and chemical and wear resistance. Modifying the block could achieve the effect of elastomer material on the mechanical, thermal, and chemical characteristics of asphalt [16]. Under the influence of high-temperature stirring, polyurethane could solidify with the highly active side groups in asphalt (such as hydroxyl, carboxyl, etc.) to promote the transformation of aromatic fractions or colloids in asphalt components to asphaltene, forming strong linked molecular chain forces and a stable spatial network, which could help improve asphalt performance [17]. Compared to other polymer modifiers, the high elasticity and durability of elastomer modifiers contributed to the improvement of asphalt’s elastic characteristics and resistance to irreversible deformation, as well as the service quality and life of the asphalt pavement [18,19,20]. The elastomer-modified asphalt mixture had superior water damage, spalling resistance, and high-temperature deformation resistance compared to matrix asphalt. However, the penetration grade of modified asphalt remained unchanged, and its resistance to low-temperature cracking was marginally lower than that of the SBS asphalt mixture [21,22]. Regarding the cracking resistance of conventional elastomer-modified asphalt at low temperatures, Zhang et al. developed a novel type of polyurethane thermoplastic elastomer asphalt mixture (PUTE) with superior low-temperature performance and water stability compared to SBS asphalt mixture, which could be applied in bridge deck pavement with excellent performance [23,24]. In addition to improving thermal and mechanical performance, the elastomer-modified asphalt could balance other attributes well. Methane-4 4’ disocyanate (MDI)-polypropylene glycol or thermoplastic polyurethane could improve the UV aging resistance of asphalt pavement materials. Adding diethylene glycol to toluene diisocyanate (TDI)-castor oil could considerably enhance asphalt’s self-healing characteristics [25,26,27]. Additionally, foamed asphalt could be created by using the theory of the isocyanate group in polyurethane reactions with water to generate a urea bond and the volatile gas CO_2_ [28,29].

In conclusion, asphalt pavement materials’ service performance and durability could be enhanced by the modification effect of the elastomer material. Many academics’ research concentrated on modified asphalt pavement materials’ thermal and antiaging properties instead of improving mechanisms. Investigating the action mechanism of the elastomer-modified asphalt could provide a precise understanding of the effect and impact of elastomer material on asphalt, which is crucial for enhancing the performance of road services. Regarding the mechanism study of elastomer-modified asphalt, the current research mainly involves the analysis of the microscopic surface structure or chemical molecular level, which lacks the mechanism exploration of the modifying effect of elastomer on asphalt, which is limited for further application of elastomer in pavement engineering. 

Therefore, as one type of typical elastomer material, polyurea was selected to prepare modified asphalt (PUA-MA) for the mechanism study. Three indexes and Brookfield viscosity tests were used to identify the physical properties of PUA-MA. The interaction state between the PUA modifier and asphalt was characterized, and the chemical element composition of the contacting microzone was examined. The thermophysical characteristics and temperature sensitivity of PUA-MA were investigated using thermogravimetric and differential scanning calorimetry tests (TG-DSC). Finally, dynamic Fourier transform infrared spectroscopy explored the evolution and behavior of chemical functional groups before and after asphalt binder modification.

## 2. Materials and Methods

### 2.1. Material and Preparation

#### 2.1.1. Asphalt

The 70# asphalt was selected as the base asphalt (BA) for preparing modified asphalt. The technical indices of 70# asphalt are shown in Table 1.

#### 2.1.2. Polyurea

Polyurea was a type of organic polymer substance generated from the block reaction between the soft and hard chains. In this article, PUA-100 was used as the raw material for preparing powder modifiers, and its critical technical performance indices were listed at Table 2.

#### 2.1.3. Preparation of PUA and PUA-MA

I. Preparation of PUA modifier

**Figure 1:**
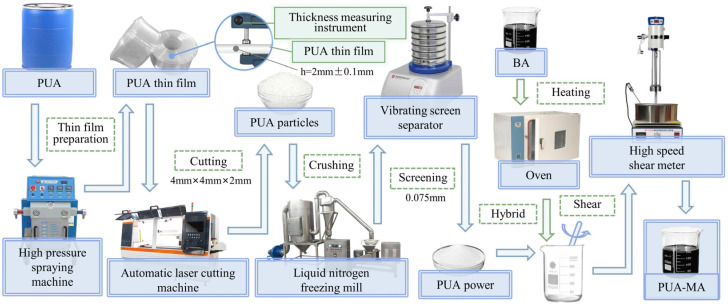
The preparation process of PUA and PUA-MA.

PUA mainly consisted of A and B components and could be formed by high-pressure spraying. The A and B components could not react perfectly to PUA if mixed with asphalt directly. Therefore, to ensure the quality of PUA material in asphalt, the PUA material would be divided into powders of good chemical stability. The preparation technique of PUA powder was performed by coupling liquid nitrogen freezing and fine grinding, and then the modified asphalt was prepared. Figure 1 depicts the processing flowchart for PUA-modified material, and the preparation process is detailed as follows:

  1. The PUA raw material of a certain weight was placed in the hopper of a high-pressure spraying machine (H20/35PUA Model, Qindao Sanhesheng Polymer Technology Co., Ltd., Qingdao, China), and the PUA film was produced under high-pressure sealing conditions. PUA film thickness should be controlled within 2 mm (±0.1 mm).
  2. The 2-mm PUA film was divided into 4–5 mm particles.
  3. The PUA particles were placed in the storage bin of the liquid nitrogen and then transferred to the mill for fine grinding.
  4. PUA powder of various particle sizes was screened, and PUA powder of 0.075 mm was chosen as the asphalt modifier.

II. Preparation of PUA-MA

A certain mass of 70# BA was weighed and heated to the molten state. The PUA modifier was weighed according to 3%, 6%, and 9% of the asphalt weight. The modifier was slowly added to the BA three times at 160 °C. After initial mixing, the asphalt with PUA was transferred to the high-speed shear instrument for high-speed shear mixing at 160 °C and 5000 rad/min for 30 min. After mixing, the mixture was placed in the oven for swelling. PUA-MA with different dosages of modifier was prepared after swelling.

### 2.2. Experimental Method

#### 2.2.1. Fundamental Performance Test

According to the standard test methods of asphalt and bituminous mixtures for highway engineering (JTG E20-2011) [30], the penetration at 25 °C, softening point, ductility at 10 °C, and Brookfield viscosity of BA and PUA-MA were tested to clarify the influence of PUA modifier on the fundamental properties of asphalt binder.

#### 2.2.2. SEM-EDS Test

SEM-EDS equipment (Hitachi SU-8220 Model, Tokyo, Japan) was utilized to characterize the microscopic morphology of the interaction state between PUA and asphalt. The test voltage was 5 kV, while the observation multiples were 100, 200, 600, 1000, 2000, and 3000. The following approaches were used for sample preparation and detection.

#### 2.2.3. TG-FTIR Test

To clarify the influence of chemical composition and structural properties of PUA-MA, TG-FTIR equipment (Nicolet IS 50 Modle, NETZSCH GMBH, Selb, Germany) was used to measure BA and PUA-MA. Nitrogen was used as the protective gas, and the test temperature range was set to 30–500 °C. The heating rate was set to 10 °C/min. The wave number range of the infrared spectrum test was set to 4000–400 cm^−1^. The resolution of the FTIR test was set to 4 cm^−1^, and the scanning time was 32.

#### 2.2.4. DSC Test

DSC equipment (DSC3+ Model, Mettler Toledo, Greifensee, Switzerland) was employed to examine the heat flow curve and heat flow value of PUA-MA. The temperature rise rate was 10 °C/min. The test temperature range was −40–200 °C. Nitrogen with a 50 m/min flow rate was used as the protective gas.

#### 2.2.5. High-Temperature Rheological Properties Test

To assess the high-temperature performance, a dynamic shear rheometer (SmartPave 102, Anton Paar company, Graz, Austria) was used to perform temperature scanning, frequency scanning, and the multiple stress creep recovery test (MSCR) on BA and PUA-MA. The temperature scan test’s temperature range was 46–76 °C (temperature interval was 6 °C). The load frequency was 10 rad/s, and the control strain was 1%. The temperature of the frequency scanning test was 60 °C. The load range was 0.1 rad/s–100 rad/s, and the control strain was 1%. The test temperature of MSCR was 64 °C. The stress levels were 0.1 kPa and 3.2 kPa. 10 s was used as a cycle. The creep duration was 1 s, and the recovery strain duration was 9 s. In the above tests, 25 mm parallel plates were used, and the gap between parallel plates was 1 mm.

## 3. Results and Discussion

### 3.1. Fundamental Performance of PUA-MA

#### 3.1.1. Physical Property

**Figure 2:**
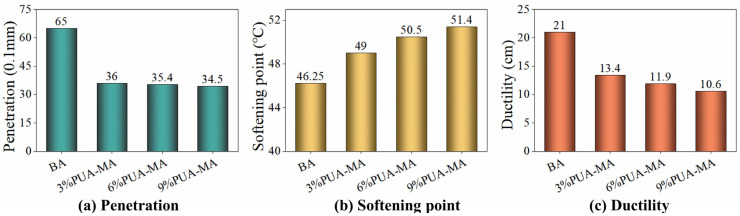
Three indicators of BA and PUA-MA ((**a**) 25 °C Penetration; (**b**) Softening point; (**c**) 10 °C Ductility).

As shown in Figure 2, the addition of PUA and the increase in dosage enhanced the softening point of PUA-MA. The softening point of PUA-MA was 49 °C when the dosage was 3%, which was 5.9% higher than that of BA. With a dosage of 6%, the softening point rose by 9.2%. With a dosage of 9%, the softening point reached a maximum of 51.4 °C, an increase of 11.1% compared to BA. Adding PUA enhanced the cross-linking between PUA and asphalt and formed a stable spatial network structure. The interactive network system prevented the thermal motion of asphalt molecules, thereby significantly enhancing the high-temperature performance of PUA-MA [31]. The inclusion of PUA decreased 25 °C penetration from 65 (0.1 mm) to 35 (0.1 mm) by a substantial amount. The penetration of PUA-MA at various dosages ranged between 53% and 55% of BA, indicating that including PUA enhanced asphalt hardening, which enhanced the asphalt’s high-temperature deformation resistance. In addition, the inclusion of PUA decreased the ductility. The ductility dropped from 15.4 cm to 10.6 cm as the modifier concentration increased. Compared to BA, the ductility of PUA-MA was decreased by 36–49%, indicating that PUA negatively influenced the asphalt’s low-temperature performance.

#### 3.1.2. Brookfield Viscosity Test

**Figure 3:**
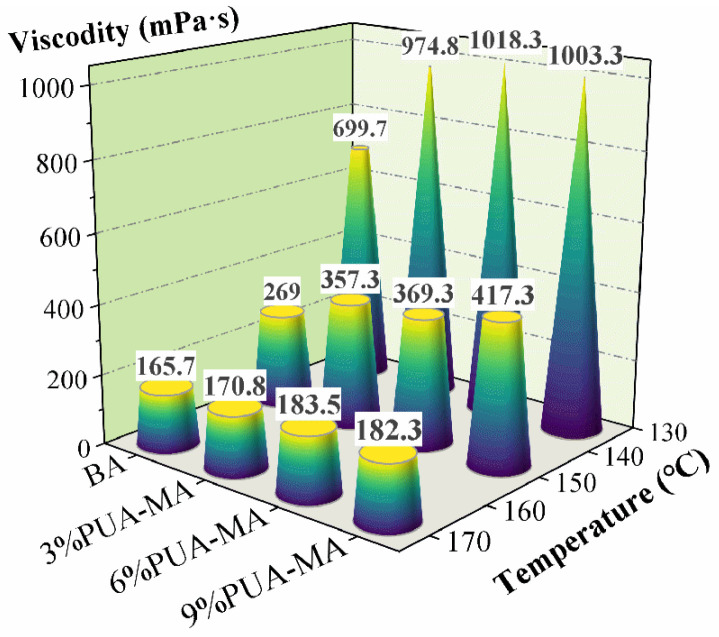
Brookfield viscosity of BA and PUA-MA.

As shown in Figure 3, the addition of PUA could improve the viscosity of asphalt significantly and the improving effect could be maintained even in temperature rising. However, the dosage of PUA could lead to different modifying effects. The viscosity of 6% PUA-MA fell from the maximum (649 mPa·s) as the temperature rose from 135 °C to 150 °C, while BA decreased the least (430.7 mPa·s). When the temperature rose from 150 °C to 175 °C, the viscosity of 9% PUA-MA decreased the most (235 mPa·s), whereas BA decreased the least (103.3 mPa·s). In addition, with the dosage increase, the viscosity at various temperatures exhibited an obvious increasing trend, showing that the increased dosage also reduced fluidity, and the high-temperature performance was greatly enhanced. When the dose was 3%, and the temperature was 135 °C, the viscosity of PUA-MA increased the most (approximately 275.1 mPa·s). The increasing viscosity range steadily diminished with an increase in dosage. The viscosity was comparable when the dose was 6% and 9%. The increased dosage of PUA raised the asphalt’s viscosity value and enhanced its performance at high temperatures.

### 3.2. Thermal Properties of PUA-MA

#### 3.2.1. TG Test

**Figure 4:**
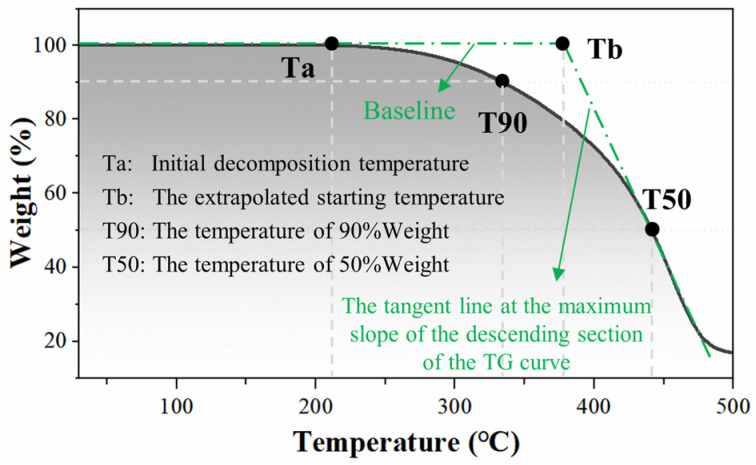
Schematic diagram of TG curve analysis index.

**Figure 5:**
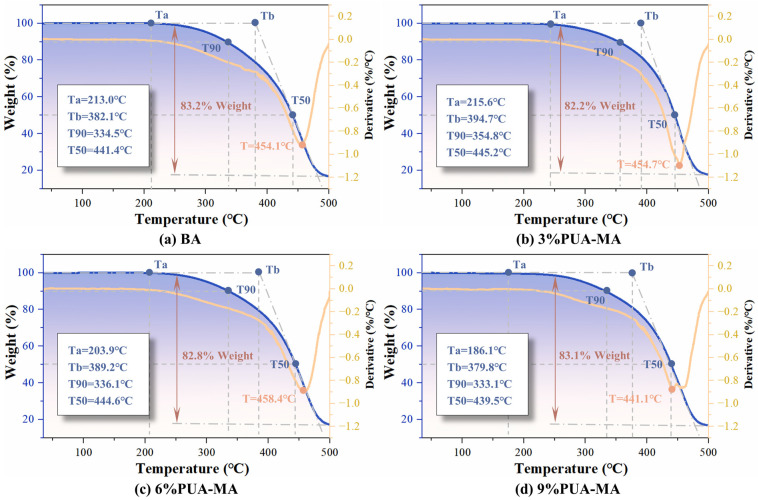
TG and DTG curves of BA and PUA-MA ((**a**) BA; (**b**–**d**) 3%, 6%, 9% PUA-MA).

The TG test indexes were determined according to Figure 4, and the results were shown in Figure 5. As seen in Figure 5, the TG and DTG curves of BA and PUA-MA within 180 °C were all straight, with a weak weight loss rate, showing that no physical and chemical reactions occurred. When the temperature rose from 180 °C to 220 °C, the TG and DTG curves of BA and PUA-MA started to decrease. The thermogravimetric phenomena happened first in 9% PUA-MA (Ta = 186.1 °C), then in 6% PUA-MA (Ta = 203.9 °C), then in BA (Ta = 213.0 °C), and lastly in 3% PUA-MA (Ta = 215.6 °C). When the temperature continued to rise, the TG and DTG curves of BA and PUA-MA showed a significant decreasing trend. At 440–460 °C, the DTG curve had a minimum value, indicating that BA and PUA-MA exhibited an evident mass loss phenomenon. At 500 °C, the total mass loss of BA, 3% PUA-MA, 6% PUA-MA, and 9% PUA-MA was 83.2%, 82.2%, 82.8%, and 83.1%, respectively. It was discovered that the overall weight loss rate of PUA-MA was less than that of BA, indicating that mass loss was reduced by adding PUA within the phase transition region.

**Figure 6:**
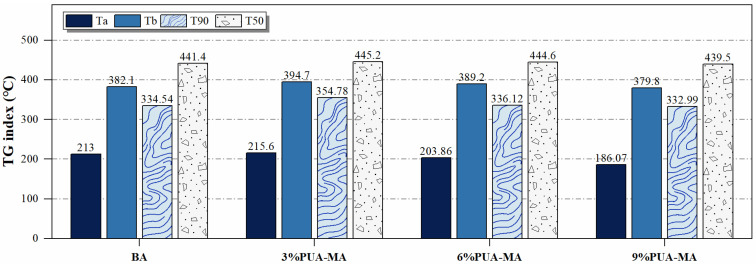
The change trend of TG index of BA and PUA-MA.

Figure 6 displayed the changing trend of TG indexes of BA and PUA-MA. Both Ta and Tb could be used to characterize the temperature of asphalt at the beginning of decomposition. As shown in Figure 6, when the dosage was 3%, Ta and Tb increased slightly. However, with the dosage increase, both indexes showed a decreasing trend. They were smaller than BA, indicating that adding PUA with a low dosage could delay mass loss, but the high dosage had no apparent effect.

T90 and T50 represented the temperature when the asphalt mass loss rate reached 10% and 50%, respectively. When the mass loss was 10%, the T90 of BA was 334.5 °C. The increase of 3% PUA-MA and 6% PUA-MA was 6.05% and 0.47%, respectively. Additionally, 9% PUA-MA (333.0 °C) was slightly lower than that of BA. When the mass loss was 50%, the T50 of BA was 444.1 °C. Furthermore, 3% PUA-MA and 6% PUA-MA were higher than BA, whereas 9% PUA-MA was marginally lower than BA. It could be seen that a modest concentration of PUA could increase the decomposition temperature of asphalt, but the effect was not significant when the concentration reached 9%. 

Due to the strong chemical bond formed between PUA and asphalt, the surface morphology of the modified asphalt changed, the polymer chain and asphalt formed a stable and high-strength network spatial structure, the mass loss of asphalt was reduced, and the thermal stability was effectively enhanced [13], among which 3% PUA-MA had the most excellent effect. It was important to note that the thermal stability enhancement effect decreases with the addition of PUA. Ta, Tb, T90, and T50 of PUA-MA were lower than those of BA when the dosage reached 9%, but the total mass loss of PUA-MA was identical to that of BA in the test temperature range.

#### 3.2.2. DSC Test

**Figure 7:**
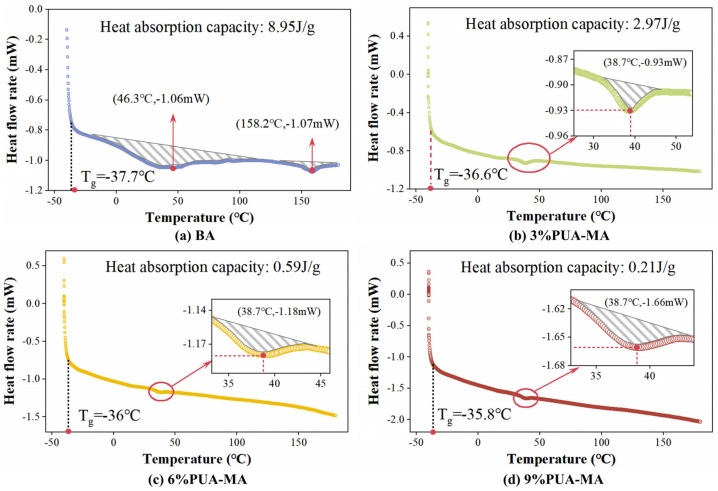
DSC curves of BA and PUA-MA.

Under constant temperature increase, the curve of the power difference between asphalt and reference with temperature change is the DSC curve, which characterizes the heat absorbed and released by the asphalt in the process of temperature increase and the change in its internal component phase structure when the temperature changes, and accurately predicts the temperature sensitivity of different asphalts. The size of the endothermic peak in the DSC curve represents the heat absorbed by asphalt during the modification of its mechanical shape [32]. The DSC curve of BA and PUA-MA is shown in Figure 7.

Figure 7 demonstrated that the BA and PUA-MA DSC curves exhibited a decreasing trend with increasing temperature. As shown in Figure 7a, the DSC curve had two endothermic peaks. BA achieved the glass transition temperature at −37.7 °C, and the structure started changing from the normal to the high elastic state. As the temperature climbed to 46.3 °C, the aggregation state of BA approached the crucial value of transformation, and the DSC curve displayed the first endothermic peak (−1.05 mW). When the temperature hit 116.7 °C, the first convergent transition stopped. BA started to undergo the second material structure change at 121.3 °C and achieved the critical value of the second transformation at 158.3 °C, where the peak value of heat absorption was −1.08 mW. In contrast, the DSC curve of PUA-MA was flat. As seen in Figure 7b–d, 3% PUA-MA, 6% PUA-MA, and 9% PUA-MA achieved the glass transition temperature at −36.6 °C, −36.0 °C, and −35.8 °C, respectively, and began to transition from the common elastic state to the high elastic state. In the endothermic stage, 3% PUA-MA, 6% PUA-MA, and 9% PUA-MA exhibited relatively modest endothermic peaks at 38 °C.

The glass transition temperature was critical in the asphalt viscoelastic state changing with temperature. The glass transition temperature of PUA-MA was found to be higher than that of BA. With increasing dosage, the glass transition temperature of PUA-MA rose. Asphalt was an amorphous material. Since macromolecules did not migrate below the glass transition temperature, asphalt was susceptible to brittle fracture at low temperatures under external force [33]. Therefore, the higher the glass transition temperature was, the worse the low-temperature performance of asphalt was, and the addition of PUA did not affect the low-temperature performance. In addition, the endothermic peak area of BA was the largest (8.95 J/g). At the same time, PUA-MA was only 0.02–0.3 times BA, indicating that the aggregation transition of BA required more energy and more components of BA change in the test temperature range, and the thermal stability performance was the worst. The addition of PUA could improve the thermal stability of asphalt.

### 3.3. Microscopic Characteristics of PUA-MA

#### 3.3.1. SEM Test 

**Figure 8:**
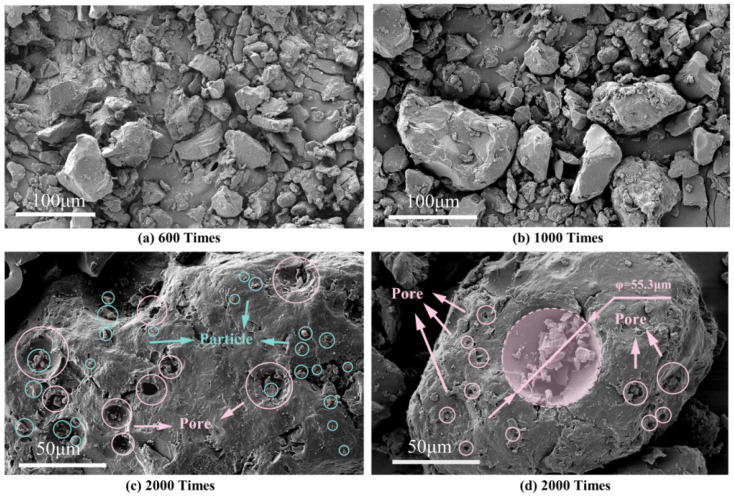
Micromorphology of PUA ((**a**) 600 times; (**b**) 1000 times; (**c**,**d**) 2000 times).

The findings of the SEM test for PUA were shown in Figure 8. At 600 times and 1000 times, the particle size of PUA particles was homogeneous and mostly diffused, with no observable agglomeration phenomena. In addition, PUA was mostly a block or sheet structure, a dense structure, or a generally flat surface. At 2000 times, a tiny number of small particles were connected to the surface of PUA particles, and there were a few pores on the surface.

**Figure 9:**
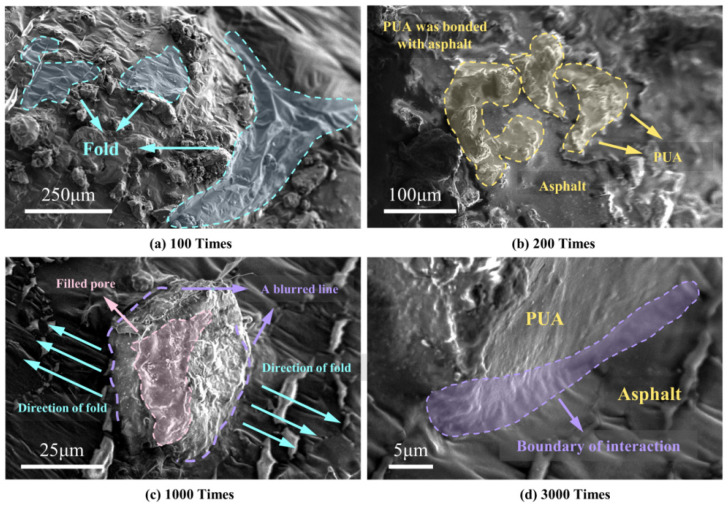
Micromorphology of PUA-MA ((**a**) 100 times; (**b**) 200 times; (**c**) 1000 times; (**d**) 3000 times).

The findings of the SEM test for PUA-MA are shown in Figure 9. At 100 times, PUA particles did not agglomerate into blocks but were generally equally spread throughout the asphalt, showing that PUA-MA is not a homogenous system but a two-phase structure. Therefore, PUA particles could not be dissolved in BA, entirely covered by asphalt as a dispersed phase [34]. In addition, the surface of PUA-MA was rough and uneven, with many tension-shaped folds. At 200 times, the surface of the PUA particles was coated with asphalt and took on a fuzzy shape.

At 1000 times and 3000 times, the surface of PUA particles was utterly coated with asphalt. Where the PUA encountered the asphalt, an irregular contact interface was formed, and tensile pleats formed in the direction perpendicular to the contact interface. The above phenomena indicated that PUA and BA had good physical compatibility. In addition, chemical reactions might occur between PUA and asphalt. Combining chemical bonds could increase asphalt and PUA’s chemical compatibility, thereby enhancing asphalt’s efficacy as a pavement material [34]. 

#### 3.3.2. EDS Test

**Figure 10:**
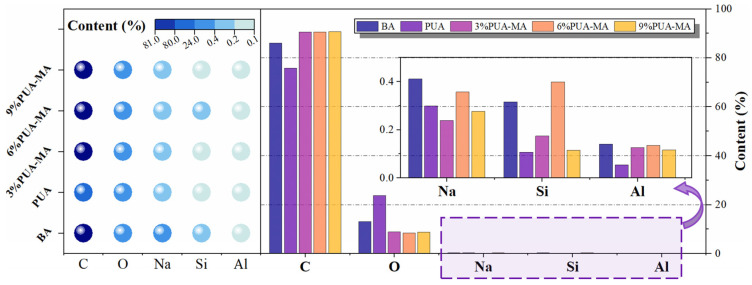
Elements contents of BA, PUA, and PUA-MA.

Figure 10 shows the EDS test results of PUA, BA, and PUA-MA. The content of the C element was the highest (75–91%), whereas the concentration of element O was 8–23%. The quantity of Na, Si, and Al elements was negligible (0.1–0.5%). Figure 10 shows that the contents of C and O in BA were 86.02% and 13.11%, respectively, and the contents of C and O in PUA were 75.79% and 23.75%, respectively. Compared with BA, the content of C in PUA-MA increased by 5.2–5.4%, and the content of O decreased by 32–35%. There was no significant difference in the concentrations of C and O in PUA-MA with different dosages. Asphalt was a complex combination of hydrocarbons with varied molecular weights and their nonmetallic derivatives. In contrast, PUA was an organic polymer substance generated by the repetitive organization of an isocyanate group (-NCO) [35]. The free isocyanate group (-NCO) in PUA combined with the hydroxyl and amino groups in asphalt (asphaltene and gum) to generate the carbamate group. Therefore, after applying the PUA modifier, the amount of C in PUA-MA rose [13,14,15,16,17,18,19,20,21,22,23,24,25,26,27,28,29,30,31,32,33,34,35,36]. On the other hand, with the addition of PUA, PUA-MA with varying dosages exhibited a low level of O element. This might be due to the chemical interaction between PUA and carboxylic acid (COOH) in asphalt, which produced volatile CO_2_ and lowered the O element content in PUA-MA [37].

#### 3.3.3. FTIR Test

**Figure 11:**
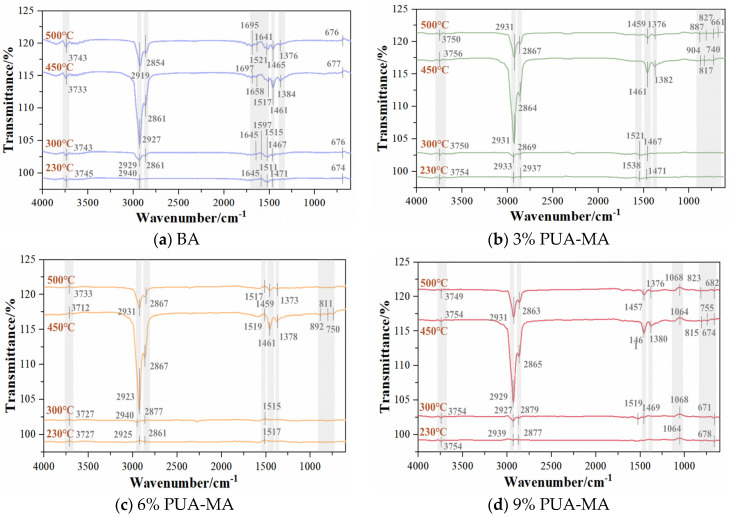
FTIR of BA and PUA-MA ((**a**) BA; (**b**) 3% PUA-MA; (**c**) 6% PUA-MA; (**d**) 9% PUA-MA).

Figure 11 showed the relationship between chemical functional groups and temperature in BA and PUA-MA. As depicted in the figure, the FTIR of PUA-MA was comparable to that of BA, indicating the presence of the physical modification processed in the PUA-MA [38]. The most substantial absorption peak existed at 2850–2950 cm^−1^, corresponding to the stretching vibration absorption peak of C-H in the -CH_2_ and -CH_3_ groups, with the -CH_2_ group being the strongest. The absorption peak with the second intensity was located at 1375–1697 cm^−1^, with multiple continuous absorption peaks with different intensities, which was the coupling effect of C=C in aromatic hydrocarbons and C-H in -CH_2_ and -CH_3_. 1517–1697 cm^−1^ was the vibration absorption peak of the benzene ring’s conjugate double bond C=C skeleton. 1461–1465 cm^−1^ was the in-plane bending vibration absorption peak of C-H in -CH_2_. 1375–1385 cm^−1^ was the in-plane bending vibration absorption peak of C-H in -CH_3_. The absorption peak of 3730–3755 cm^−1^ was the stretching vibration peak of the -OH group, and 660–675 cm^−1^ was the fingerprint region of BA, which was caused by the out-of-plane rocking vibration of C-H on the benzene ring. 1055–1065 cm^−1^ and 660–900 cm^−1^ were the fingerprint regions of PUA-MA, in which 1055–1065 cm^−1^ were the stretching vibration absorption peaks of C-O, C-N, and C-C, and 660–900 cm^−1^ were the deformation vibration absorption peaks of C-H, N-H, and O-H. 

Generally, at 230–450 °C, the absorption peaks at various positions were enhanced, indicating that the thermal stability of BA and PUA-MA was good. At 500 °C, the absorption peak intensity diminished at 2854–2940 cm^−1^ (C-H of alkanes or cycloalkanes) and 1375–1465 cm^−1^ (C-H of -CH_3_ and -CH_2_), indicating the thermal decomposition of C-H in BA and PUA-MA. The above position’s absorption peak area was calculated. When the temperature increases from 450 °C to 500 °C, the absorption peak area decreased by 61.3%, 65.5%, 57.7%, and 54.5% for BA, 3% PUA-MA, 6% PUA-MA, and 9% PUA-MA, respectively, at 2854–2940 cm^−1^. At 1375–1465 cm^−1^, the absorption peak area reduction rates for BA, 3% PUA-MA, 6% PUA-MA, and 9% PUA-MA were, respectively, 67.1%, 72.8%, 71.6%, and 71.3%. It could be observed that the higher the dosage, the smaller the diminution rate of the absorption peak area, and the more stable the network structure formed between PUA and asphalt so that PUA-MA was resistant to thermal decomposition.

**Figure 12:**
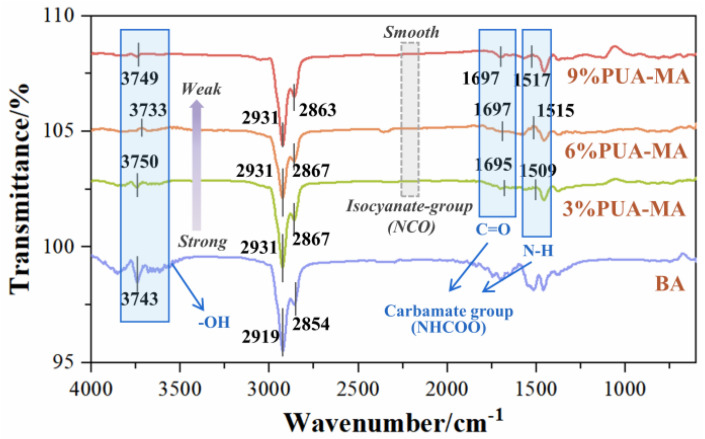
FTIR of BA and PUA-MA at 500 °C.

The characteristic functional group analysis was performed based on the results of 500 °C. As seen in Figure 12, 3730–3750 ccm^−1^ was the hydroxyl (-OH) absorption peak. It could be found that the hydroxyl absorption peak area of PUA-MA was obviously smaller than that of BA, approximately 0.12–0.23 times BA. In addition, the presence of an absorption peak was detected at 1517 cm^−1^ for PUA-MA, which corresponded to the bending vibration absorption peak of N-H in carbamate (-NHCOO), as well as the weak absorption peak at 1695–1697 cm^−1^, which corresponded to the stretching vibration absorption peak of C=O in NHCOO. No isocyanate group was identified at approximately 2273 cm^−1^ of PUA-MA, suggesting that the isocyanate group in the PUA modifier interacted with the asphaltene hydroxyl group (OH) to produce the carbamate group (-NHCOO) [13]. In conclusion, both physical modification and chemical modification processes occurred inside PUA-MA, which help in achieving the modifying effect on asphalt binder. Meanwhile, the physical modification might be the main cause of low temperature property degradation of PUA-MA.

### 3.4. High-Temperature Rheological Properties of PUA-MA

#### 3.4.1. Temperature Sweep Test

**Figure 13:**
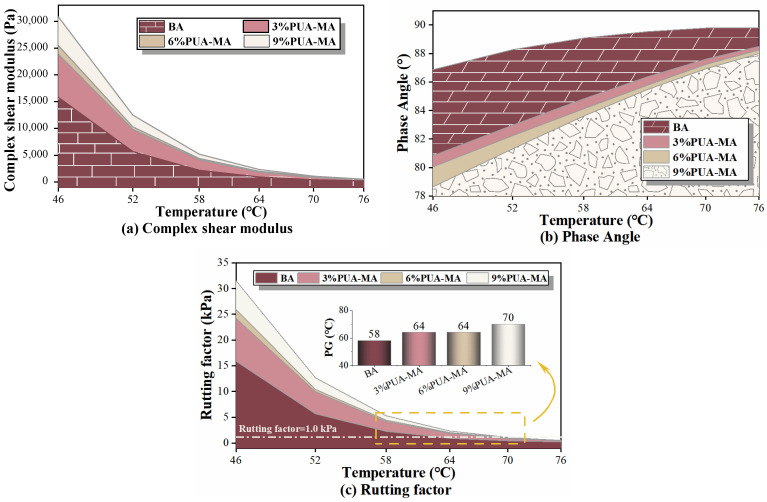
Temperature scanning test indexes of BA and PUA-MA ((**a**) complex shear modulus; (**b**) phase angle; (**c**) rutting factor).

The results of the BA and PUA-MA temperature scanning tests at high temperatures (46–76 °C) are shown in Figure 13. Complex shear modulus and rutting factor were indexes to characterize the high-temperature deformation resistance of asphalt. At the same time, the phase angle was an index to measure the ratio of viscous and elastic components in asphalt. The more elastic the components of asphalt were, the greater their capacity for elastic recovery and resistance to rutting at high temperatures. This was shown by the bigger complex shear modulus and rutting factor and the lower phase angle [39].

According to Figure 13, the complex shear modulus and rutting factor of BA and PUA-MA with various mixing quantities dropped as the temperature rose, but the phase angle rose. When the temperature increases to 70–76 °C, the complex shear modulus and rutting factor of PUA-MA had no significant difference with that of BA, indicating that the modifier had a significant effect on the viscoelasticity of asphalt. Due to the interaction between terminal isocyanate in PUA modifier and polar groups in asphalt, PUA and asphalt cross-linked and formed a stable three-dimensional network structure, which inhibited the transition of asphalt to viscous flow state within a certain range (the complex shear modulus and rut factor of PUA-MA were higher than BA). At exceedingly high temperatures, however, the asphalt’s molecular motion progressively became more intense, the degree of cross-linking between particles decreased, and the network structure was gradually destroyed. Consequently, the inhibitory effect of the PUA modifier dissipates (the complex shear modulus and rutting factor of PUa-Ma had no discernible difference from BA), the viscous component of asphalt increased, and the elastic component decreased. Therefore, asphalt’s high-temperature deformation and rut resistance were diminished [40].

Compared with BA, PUA-MA significantly increased the complex shear modulus and rut factor by 1.5–2 times and reduced the phase angle by 6–8.2°. With the increase in modifier content, the complex shear modulus and rut factor showed a continuous increase, while the phase angle showed a continuous decrease. Asphalt’s complex shear modulus and rutting factor increased by 1603 Pa and 1.7 kPa, respectively, when the content increased from 3% to 6%, while the phase angle decreased by 0.8°. As the content increased from 6% to 9%, asphalt’s complex shear modulus and rutting factor increased by 5384 Pa and 1.4 kPa, respectively, while the phase angle decreased by 5.6°. According to the asphalt performance grading standard proposed by SHRP, asphalt pavement performance grades (PG) were divided according to rut factor ≥1.0 kPa, as shown in the bar chart of Figure 13c. The figure showed that the PG grading of PUA-MA was higher than that of BA (58 °C) and increased with the dosage increase. Asphalt had the highest PG grade (70 °C) when the dosage was 9%. The data mentioned above indicated that PUA had a significant effect on improving the high-temperature performance of asphalt. The addition of PUA and an increase in its dosage considerably enhanced the asphalt’s elastic properties, as well as its resistance to rutting and inability to recuperate at high temperatures. The modification effect was most pronounced at a concentration of 9%.

#### 3.4.2. Frequency Scanning Test

**Figure 14:**
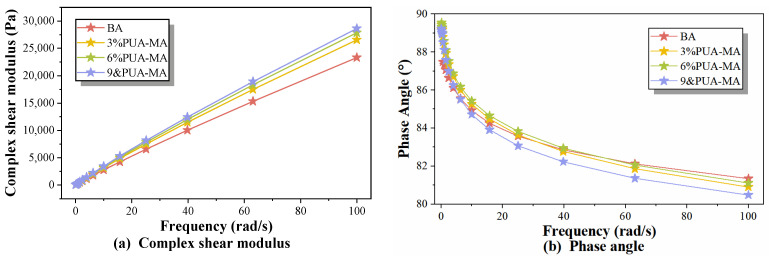
Frequency scanning test index of BA and PUA-MA ((**a**) complex shear modulus; (**b**) phase angle).

At 60 °C, a frequency scanning test was conducted for BA and PUA-MA with varying dosages to simulate the impact of driving speed on asphalt pavement, with low angular frequency representing low speed and high angular frequency representing high speed. Figure 14 depicts the results. 

The complex shear modulus of BA and PUA-MA increased with increasing angular frequency, as shown in Figure 14a. This was because the greater the angular frequency, the greater the number of times the asphalt was subjected to vibration per unit of time. The lesser the strain generated the greater the resulting complex modulus. Consequently, the complex shear modulus of areas with low angular frequency (low speed) was less than that of areas with high angular frequency (high speed). Therefore, potholes were more likely to form when slow-moving heavy-duty vehicles interacted with asphalt pavement. This was also why channeled traffic, such as parking lots, low-speed highways, and emergency brakes, was more likely than expressways to cause pavement diseases [41]. When asphalt was in the low-frequency zone (0.1–25 rad/s), the complex shear modulus of BA did not differ substantially from that of PUA-MA. When the asphalt was in the high-frequency zone (80–100 rad/s), the complex shear modulus of PUA-MA was significantly higher than that of BA (approximately 1.1–1.2 times of BA) and increased with the increase in the modifier content, indicating that the modification effect of PUA modifier on the low-speed highway was limited, but had a significant effect on the improvement of the anti-rut performance of the highway. When the amount of PUA modifier increased, the three-dimensional network structure formed by the cross-linking of PUA molecules and asphalt molecular chains became more stable. The asphalt’s performance at high temperatures improved substantially [42]. The enhancement effect of asphalt high-temperature performance was greatest when the PUA modifier content was 9%.

As shown in Figure 14b, the phase angles of BA and PUA-MA decreased as the angular frequency increased, indicating that the increase in angular frequency improved the elasticity of asphalt and its resistance to irreversible deformation. When the angular frequency was less than 40 rad/s, the phase angle of PUA-MA was greater than BA. When the angular frequency was greater than 40 rad/s, the phase angles, from largest to smallest, were BA, 6% PUA-MA, 3% PUA-MA, and 9% PUA-MA, indicating that the addition of PUA improved the elastic recovery ability of asphalt at medium and high frequencies. When the modifier content was 9%, asphalt had the greatest elasticity. 

#### 3.4.3. MSCR Test

**Figure 15:**
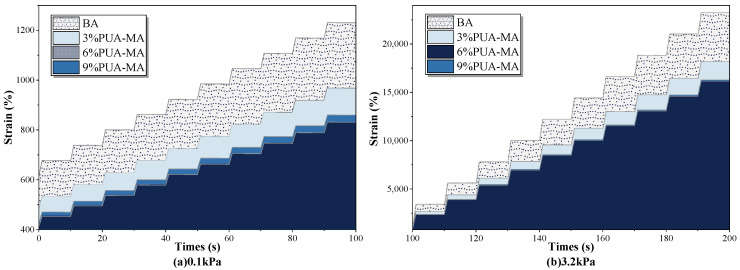
Strain-time curves of BA and PUA-MA ((**a**) 0.1 kPa; (**b**) 3.2 kPa).

**Figure 16:**
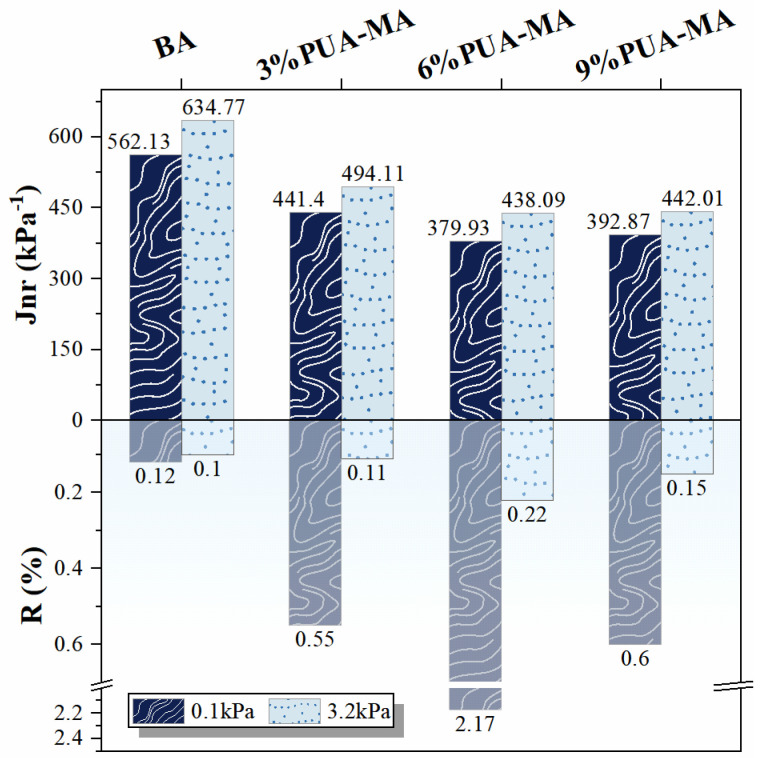
Jnr and R of BA and PUA-MA.

To determine whether there was an elastic response of PUA-MA and the change rule of the elastic response under two different stress levels, MACR tests at 64 °C were conducted on BA and PUA-MA with different dosages, and the results were shown in Figure 15 and Figure 16.

As shown in Figure 15, the cumulative strain of BA and PUA-MA increased as the loading duration increased, and the cumulative strain of asphalt at a 3.2 kPa stress level was greater than that at a 0.1 kPa stress level. Compared to BA, the cumulative strain of PUA-MA decreased by 21.5% to 32.5% at various concentrations, and the greater the modifier, the lower the cumulative strain. For a stress level of 0.1 kPa, the cumulative strain increased from 0 s to 100 s by 610%, 481%, 416%, and 428% for BA, 3% PUA-MA, 6% PUA-MA, and 9% PUA-MA, respectively. When the time increased from 100 s to 200 s at 3.2 kPa, the cumulative strain increased by BA, 3% PUA-MA, 6% PUA-MA, and 9% PUA-MA were respectively 21,844%, 17,085%, 15,179%, and 15,307%. In conclusion, PUA-MA was less sensitive to changes in stress level, and the addition and dosage of PUA modifier considerably improved the asphalt’s elastic recovery capacity.

To further investigate the elastic resilience of PUA-MA, the non-recoverable creep compliance (Jnr) and creep recovery rate (R) were calculated based on the data in Figure 15, and the results were depicted in Figure 16. Jnr was an index that measured the asphalt’s resistance to persistent deformation under repeated load. The smaller Jnr, the greater asphalt’s resistance to rutting [43,44]. Figure 16 demonstrated that the Jnr of BA and PUA-MA at 3.2 kPa stress level was less than Jnr at 0.1 kPa stress level, indicating that it was harder for asphalt to recover deformation under heavy load [45]. At the same stress level, with the addition of PUA and increased the content, Jnr showed a decreasing trend (decreased by 120–196 kPa^−1^), indicating that PUA-MA had a significant enhancement effect on the rut resistance and deformation resistance of asphalt. When the content was 6–9%, PUA-MA had the strongest high-temperature deformation resistance. R was the asphalt delay elasticity index. The greater the value of R, the greater the asphalt’s elastic recovery and deformation resistance [45]. Figure 16 demonstrated that the R of asphalt at 3.2 kPa stress level was 1–10 times greater than that at 0.1 kPa stress level. The greater the stress level, the lower the R. This was because heavy vehicles created deeper ruts on the actual road surface and became permanently deformed and difficult to recover from. At the same stress level, the R of PUA-MA was significantly higher than that of BA (0.12%), and 6% PUA-MA had the largest R (2.17%), indicating that after adding PUA, the elastic recovery ability and deformation resistance of asphalt were significantly enhanced, and 6% PUA-MA had the strongest elastic deformation ability. Notably, when the stress level was 3.2 kPa, the R of BA and PUA-MA ranged from 0.1% to 0.2%, indicating that BA and PUA-MA basically lost their elastic recovery ability at high-stress levels. In contrast, their creep recovery ability was stronger at low-stress levels.

To sum up, the addition of PUA and the increase in dosage promoted the physical and chemical reactions between the isocyanate group in PUA and the hydroxyl and carboxyl groups on the surface of asphaltene, forming stable hydrogen bonds and chemical bonds, thereby increasing the compatibility of PUA modifier and asphalt [46]. The rutting resistance, deformation resistance, and elastic recovery ability of PUA-MA were also substantially enhanced, with a concentration of 6–9% producing the best high-temperature asphalt performance.

## 4. Conclusions

The conclusions of study were shown as follows:

1. The particles of the PUA modifier were mainly massive structures with dense structures and a small number of surface apertures. After being coated by asphalt, the particles formed an infiltration state with excellent physical compatibility.
2. PUA substantially improved the thermal stability of asphalt, thereby increasing the thermal decomposition temperature and decreasing the thermal mass loss. However, the glass transition temperature and low-temperature performance were diminished.
3. With the addition of PUA and the increase of dosage, softening point and Brookfield viscosity of asphalt were considerably enhanced, as was its performance at high temperatures. However, asphalt’s penetration and elongation decreased, and its low-temperature efficacy was not enhanced.
4. PUA modifier significantly improved the rheological properties of asphalt at high temperature. Increasing complex shear modulus, rut factor, PG, and R, and reducing phase Angle and Jnr could enhance asphalt’s rutting resistance at high temperature. The higher the dosage of the modifier, the better the performance.
5. Although the high-temperature performance and thermal stability of asphalt were the best when the content of PUA was 6–9%, the low-temperature performance of asphalt was not improved effectively at high content. Therefore, the recommended content of the PUA modifier was 6%.

## Figures and Tables

**Table 1:** Technical indices of 70# asphalt.

Technical Indices		Measured Value	JTG E20-2011 [30]
Softening point/°C		49	44–54
Ductility (10 °C, 5 cm/min)/cm		>100	≥100
Penetration (25 °C, 100 g, 5 s)/0.1 mm		67.8	60–80
Brookfield viscosity (135 °C)/mPa·s		699.7	—
PG grading		58	—
TFOT Residue	Mass change/%	0.2	≤0.8
	Residual penetration ratio/%	70	≥61
	Residual ductility (10 °C)/cm	18	≥15

**Table 2:** Technical performance indices of the PUA-100.

Type	Solid Content/%	Viscosity/cps	Tensile Strength/MPa	Tensile Strength/%
PUA-100	81–85	≤800	28	375

## Data Availability

Data are contained within the article.
